# Adaptations of an RCT during COVID: Case Study in one Rural American Indian Community

**DOI:** 10.33596/coll.86

**Published:** 2022-01-20

**Authors:** JESSICA D. HANSON, KYRA OZIEL, AMY HARRIS, MICHELLE SARCHE, MARCIA O’LEARY, DEDRA BUCHWALD

**Affiliations:** University of Minnesota Duluth, US; Washington State University, US; University of Minnesota Duluth, US; University of Colorado Anschutz Medical Center, US; Missouri Breaks Industries Research Inc, US; Washington State University, US

**Keywords:** COVID-19, Indigenous communities, program adaptations, alcohol-exposed pregnancies, randomized controlled trials

## Abstract

The COVID-19 pandemic is global in nature but especially threatens American Indian and Alaska Native (AI/AN) communities due to pre-existing conditions and social determinants of health. Because of the higher risk to AI/AN communities, many tribal nations have been proactive in their policies to keep the virus at bay, including travel restrictions and lockdowns. This affected tribal programs as well as collaborative research projects. One project impacted is the Native CHOICES project, an ongoing randomized controlled trial with an AI/AN community that is focused on the prevention of alcohol-exposed pregnancies. Originally designed to be conducted via in-person motivational interviewing sessions, COVID-19 restrictions precluded the intervention from being delivered in-person as it was designed. The study team received valuable input from the project’s Community Advisory Board (CAB) and community-based staff to establish a feasible and acceptable way of conducting the intervention while respecting tribally-enacted COVID-19 restrictions. The goal of this brief report is to outline not just the process to adapting to COVID-19 but also to provide recommendations for future public health programs, including the ongoing need to consider gaps in access affecting resource-poor settings.

American Indian and Alaska Native (AI/AN) communities in the United States face a wide variety of health disparities, largely due to limited access to healthcare compared to the general population owing to healthcare systems that are underfunded and under-resourced, racism and discrimination in healthcare, a dearth of culturally-attuned services, and other access barriers such as transportation. Many AI/AN communities have therefore partnered with academic institutions to develop, implement, and evaluate public health interventions that address various health disparities. These efforts are often funded by grants both at the regional and federal level in the U.S., and usually involve components of community-based participatory research.

More recently, the focus of public health interventions have shifted to the COVID-19 pandemic, which is global in nature but especially threatens AI/AN communities. According to Hatcher and colleagues (2020), “the cumulative incidence of laboratory-confirmed COVID-19 among AI/AN persons was 3.5 times that among non-Hispanic white persons” (p. 1169). There are fewer ICU beds in rural and reservation communities compared to urban areas, so AI/AN patients suffering severely from COVID-19 may not have immediate access to the necessary medical attention. Other structural inequities have contributed as well, such as inadequate housing that leads to crowded living conditions where social distancing is difficult, lack of running water for sanitizing, lack of reliable sources of electricity that make long-term food storage possible, and limited community resources such as grocery stores that can also become crowded when few are available (Arrazola et al., 2020). These disparities reflect pre-existing conditions that have made AI/AN communities particularly vulnerable to the most severe impacts of the virus.

Because of the higher risk to AI/AN communities, many tribal nations have been proactive and aggressive in their public health policies to keep the virus at bay. As sovereign nations, tribes have often imposed stricter regulations to prevent the spread of COVID-19 when compared to surrounding communities. These policies have included closing reservation borders to non-tribal members, travel restrictions, and lockdowns of various types. Figure 1 outlines the actions that one tribe took to control the spread of COVID-19 in their community. Local or state governments did not always support these types of policies. For example, tribes in one state were asked to remove travel limitations and checkpoints on roads within reservation boundaries or face the withholding of government aid and contracts. Alongside local measures to ensure the safety of AI/AN communities, various resources were developed specifically for tribal use, including culturally tailored materials such as infographics, fact sheets, radio PSAs, and social media posting materials.

The success of these prevention efforts are yet to be seen, but in general, other public health prevention campaigns and interventions have had to adjust as focus has shifted to COVID-19. In particular, many public health programs have modified their mode of delivery so that program staff and clients can remain safe from the virus while still providing and receiving valuable services; often, this meant that services that were usually provided in person shifted to being provided remotely, using telehealth and other technology. University-community partnerships and research projects funded by regional and federal governments have been challenged to adapt so that they too can remain operational yet safe. The goal of this paper is to provide documentation of how one university-tribal partnership pivoted an in-person intervention to be completed remotely, and how the input of the community was invaluable to maintaining the momentum of the recruitment into the program. As well, the paper provides an overview of how future interventions can be adapted to reflect the lessons provided by the COVID-19 pandemic.

## IMPACT ON PUBLIC HEALTH PROGRAMS: LOSS OF IN-PERSON INTERACTION

One university-tribal partnership impacted by the COVID-19 pandemic is between researchers at Washington State University (WSU) and a tribe in the Great Plains of the United States. The tribe and the WSU have worked together for a decade on multiple federally-funded studies. These studies have grown out of well-established relationships that entailed many conversations, emails, and telephone calls to ensure that public health interventions align with the tribe’s culture, needs, and service ecology. Input into these interventions is sought from project Community Action Boards (CAB), comprised of individuals from the study population, local elders, community leaders, and health care providers. Each project’s CAB advises and supports studies, assist in refining interventions as needed, and helping to disseminate study-related information.

One of these WSU-tribal partnerships is an ongoing randomized controlled trial (RCT) called Native CHOICES. The focus of this RCT is on the prevention of alcohol-exposed pregnancies (AEP) with preconceptional AI/AN people. The RCT utilizes the evidence-based CHOICES intervention, which is designed for delivery via in-person motivational interviewing sessions to support reducing risky drinking and increasing effective contraceptive use. Tribal communities previously adapted CHOICES, and an earlier efficacy study showed significantly reduced AEP risk among AI/AN (Hanson et al., 2017). Native CHOICES was designed to build on these promising findings by implementing an RCT of the adapted CHOICES intervention with AI/AN residing in the tribal community in partnership with WSU.

Recruitment and enrollment for Native CHOICES began in 2019, and monthly recruitment goals were met until March 2020, when COVID-19 restrictions precluded the intervention from being delivered in-person as it was designed. The study team utilized the project’s CAB to determine a feasible and acceptable way of conducting the intervention while respecting tribally-enacted COVID-19 restrictions. Input from community-based staff and our CAB indicated that videoconferencing (e.g. Zoom) was only possible if the study could provide access to Wi-Fi, as many community members cannot afford to pay for Wi-Fi and any available Wi-Fi is unreliable. To meet this need, our community-based research partner created an enclosed space outside of their building so participants could access Wi-Fi and therefore still participate in the RCT.

While the technology gap was theoretically bridged, the study team had to rethink recruitment and enrollment strategies again in November 2020 due to rapidly rising rates of COVID-19, which led to a tribally-enforced lockdown, as well as the impending winter weather that would limit the feasibility of using an outdoor space. This lockdown was expanded to completely limit movement on reservation lands in December 2020: only tribally approved “runners” could travel to pick up and deliver groceries and other emergency supplies. As the lockdowns and the winter weather were not conducive to our participants accessing the Wi-Fi spot set-up by the community research partner, our study team worked with local staff to conclude that all data collection would be done over the telephone until conditions improved. There have been only a few examples of CHOICES and similar interventions conducted via remote means (Symons et al., 2018), including over the telephone (Hanson et al., 2013).

Therefore, participants in Native CHOICES received their intervention materials electronically, by mail, or through contactless pick-up and will continue to do so until in-person interventions are possible. The introduction of the COVID-19 vaccination would theoretically make completing Native CHOICES in-person feasible, but slow uptake of the vaccination among rural communities as seen across the nation, as well as the increase of COVID-19 variants, means this university-community partnership continues remotely. Participation in Native CHOICES will continue to be conducted entirely over the telephone, with data entered into the online REDCap system. For the duration of COVID-19 restrictions, the study has permission to implement a modified form of verbal consent where participants provide both verbal consent as well as a supplemental electronic message.

## IMPACT ON PUBLIC HEALTH PROGRAMS: RETURNING TO IN-PERSON

Reverting the Native CHOICES to an in-person intervention while the COVID-19 pandemic continues must be done carefully and with proper community input and approval. While states, businesses, and communities “open up,” many research projects are ramping up in-person recruitment and enrollment. Research with tribal communities is at a turning point, however, as decisions are made about starting in-person research projects again. No university-community partnership has been in such a position in recent memory; therefore there is no precedence as to who makes the final decision to restart research with tribal communities. It’s likely that the COVID-19 pandemic will have lasting impacts on public health programming at the community level, and many lessons have been learned in moving forward with future endeavors. In particular, tribal communities, which have a convoluted history with research, healthcare, and non-Native partners, must lead the way in how local public health programs emerge post-pandemic.

First, one must consider tribal sovereignty; the authority of tribal nations to self-govern is affirmed in hundreds of treaties and rulings from all three branches of the U.S. government (National Congress of American Indians, n.d.). Local tribal governments have the authority to create their own policies in public health emergencies (Hershey, 2019), and there are recommendations that tribal, state, and local governments can undertake to converge coordinated planning for public health emergencies, such as the COVID-19 pandemic (Barnard, 2015). However, as seen in the current pandemic and the reaction of states to tribal closings and roadblocks, there is still jurisdictional uncertainty that has only amplified tribal-state tension during the pandemic, as highlighted earlier (Barnard, 2015). This historical tension, coupled with problematic research that has taken place on tribal lands, has led to “a narrative around collective protection, collaborative research partnerships, and tribal sovereignty over research” (O’Keefe, 2019, p. 172), which ultimately can impact existing university-tribal collaborations. The Native CHOICES intervention continues to adhere to tribal policies regarding pandemic-related closures and recognizes that tribal sovereignty supersedes any needs of the grant.

This movement of tribal sovereignty over research—specifically the role of tribal councils or tribally-run research review boards—is the second item that must be considered when restarting recruitment of participants during the COVID-19 pandemic. Many tribes have their own institutional review boards (IRBs) that review any projects prior to implementation on tribal reservation lands (Kuhn et al., 2020). This tribal oversight might be at the tribal nation level (e.g. tribal council or tribal government), at tribally-run colleges or universities, at Indian Health Service (IHS) facilities, or via tribally-based research oversight, which is typically an IRB committee at the local tribal health department level (Around Him et al., 2019). Sometimes these IRBs or ethics committees are intertribal, meaning they include multiple individual tribes or reserves under one umbrella (Hiratsuka et al., 2017; Kelley et al., 2013). Regardless of the structure, these tribal IRBs oversee a local review process through the lens of tribal priorities and sovereignty (Hiratsuka et al., 2017; Morton et al., 2013).

In the case of Native CHOICES, the project had approval from three different IRBs prior to the in-person study starting: the lead investigator’s university IRB, the IRB for the area IHS, and tribal council approval, which is a multi-step process. When moving to remote, the university-tribal partners first obtained approval from the project’s CAB and then proceeded to submit the revised protocol to the tribe and to the other two IRBs. Additional tribal approval has been sought to revert back to in-person, but at the time of this manuscript development, that approval has not been secured. The majority of tribes have a clear approval process for any new or ongoing research project. In the case of Native CHOICES, both the university and IHS IRB have stated they will defer to the tribal decision, meaning that tribal sovereignty is both recognized and respected.

## LESSONS LEARNED

Pandemic-related lockdowns and technology limitations dictated a need to provide this public health program in ways that were feasible regardless of best practice guidelines. The university-tribal partnership was fortunate to have a strong CAB to guide our adaptation efforts and provide information about the broader political and social contexts in which the intervention was occurring. The collaborative team was able to adequately adjust the Native CHOICES intervention by continually considering gaps in accessing technology and feasibility given current resources. In future studies, it may be necessary to provide broadband access via hotspots for more equitable access. The Native CHOICES intervention was funded through a federal grant, which limited the opportunity to fund such resources, therefore alternative ways of providing public health to rural communities should be considered at a much earlier stage. Finally, while technology access continues to be a challenge for public health interventions in rural communities, including tribes, public health professionals must both understand and respect where community staff and participants are in terms of access and priorities. The Native CHOICES partnership was a continuation of an existing relationship between a university (WSU) and a tribe, which benefited the program as it shifted to a remote format. The use of continued community input was essential in developing an approach that was both feasible and acceptable for ensuring ongoing access to this critical public health intervention.

In conclusion, once tribal and other communities begin to “reopen” after the COVID-19 pandemic, universities must work closely with their community partners in implementing public health research programs as they were originally intended. As noted, the Native CHOICES program deferred to the tribal approval process, which will dictate what can proceed and when. This is the first step in acknowledging tribal authority and paving the way for a respectful ongoing collaborating with AI/AN communities. Previous research has emphasized the importance of tribal sovereignty and a tribally-run research approval process in maintaining community control of resources and interests, which ultimately leads to better public health research programs (Oetzel et al., 2015). Because the global pandemic has created a situation that has no precedence and therefore no easy answers, researchers must work closely with tribal and other community partners to decide when and how research programs will be recruiting and enrolling participants in person again.

## Figures and Tables

**Figure 1:**
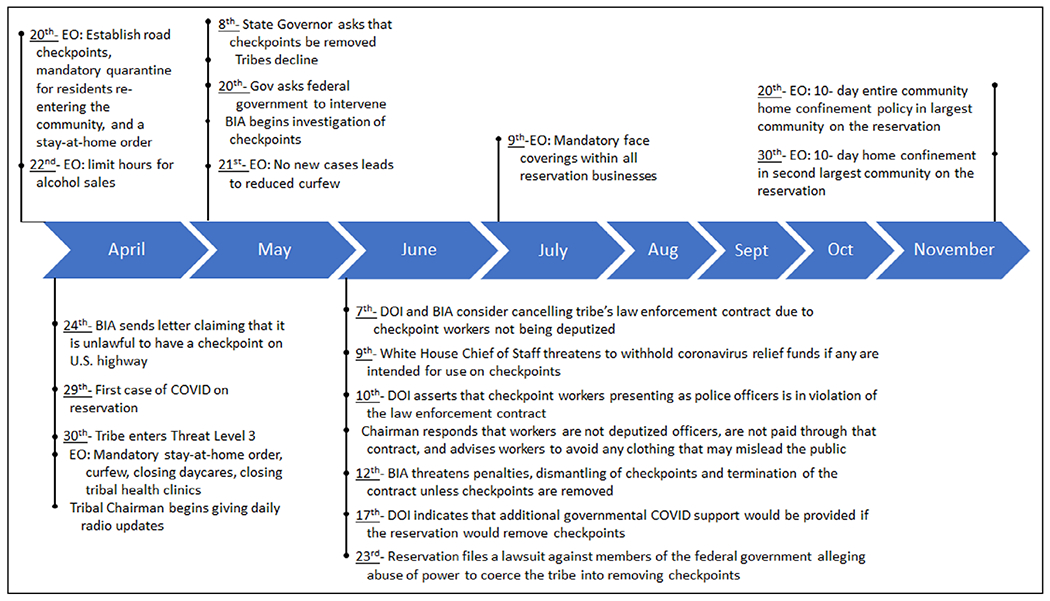
One Tribe’s Response to COVID-19. Notes: 1. All months represented are in the year 2020. 2. Acronyms: EO = Executive Order from Tribal Coucnil; BIA = Bureau of Indian Affairs; DOI = Department of the Interior.
